# Maturation of the internal auditory canal and posterior petrous bone with relevance to lateral and posterolateral skull base approaches

**DOI:** 10.1038/s41598-022-07343-9

**Published:** 2022-03-03

**Authors:** Robert C. Rennert, Michael G. Brandel, Jeffrey A. Steinberg, Rick A. Friedman, William T. Couldwell, Takanori Fukushima, John D. Day, Alexander A. Khalessi, Michael L. Levy

**Affiliations:** 1 Department of Neurological Surgery, University of California, San Diego, La Jolla, CA USA; 2 Division of Otolaryngology, Head and Neck Surgery, Department of Surgery, University of California - San Diego, La Jolla, CA USA; 3 Department of Neurosurgery, University of Utah, Salt Lake City, UT USA; 4 Carolina Neuroscience Institute, Raleigh, NC USA; 5 Department of Neurosurgery, University of Arkansas, Little Rock, AR USA; 6 Department of Neurosciences and Pediatrics, University of California San Diego, San Diego, CA USA; 7 Pediatric Neurosurgery Division, University of California San Diego, 7910 Frost St., Suite 120, San Diego, CA 92123 USA

**Keywords:** Anatomy, Nervous system, Brain, Paediatrics, Paediatric research

## Abstract

Anatomic knowledge of the internal auditory canal (IAC) and surrounding structures is a prerequisite for performing skull base approaches to the IAC. We herein perform a morphometric analysis of the IAC and surgically relevant aspects of the posterior petrous bone during pediatric maturation, a region well-studied in adults but not children. Measurements of IAC length (IAC-L), porus (IAC-D) and midpoint (IAC-DM) diameter, and distance from the porus to the common crus (CC; P-CC) and posterior petrosal surface (PPS) to the posterior semicircular canal (PSC; PPS-PSC) were made on thin-cut axial CT scans from 60 patients (grouped by ages 0–3, 4–7, 8–11 12–15, 16–18, and > 18 years). IAC-L increased 27.5% from 8.7 ± 1.1 at age 0–3 to 11.1 ± 1.1 mm at adulthood (p = 0.001), with the majority of growth occurring by ages 8–11. IAC-D (p = 0.52) and IAC-DM (p = 0.167) did not significantly change from ages 0–3 to adult. P-CC increased 31.1% from 7.7 ± 1.5 at age 0–3 to 10.1 ± 1.5 mm at adulthood (p = 0.019). PPS-PSC increased 160% from 1.5 ± 0.7 at age 0–3 to 3.9 ± 1.2 mm at adulthood (p < 0.001). The majority of growth in P-CC and PPS-PSC occurred by ages 12–15. Knowledge of these patterns may facilitate safe exposure of the IAC in children.

## Introduction

The dimensions and anatomic relationships of the internal auditory canal (IAC) to the components of the inner ear within the petrous temporal bone have important implications for the performance of lateral and posterolateral skull base approaches, in particular for the treatment of pathologies involving the IAC such as acoustic neuromas^1–5^. Familiarity with this anatomy is particularly important when hearing preservation is a surgical goal, such as with the retrosigmoid transmeatal and middle fossa approaches^2,3,5–8^.

In adults, numerous cadaveric and radiographic studies have defined the anatomic dimensions and variability of this region, including the size and shape of the IAC^2,9–15^, as well as the distance of the IAC to surgically relevant inner ear structures such as the posterior semicircular canal (PSC), vestibule, and common crus (CC)^2,3,13^. Descriptions of these parameters in children are nonetheless limited^16,17^, despite other known differences in skull base anatomy between children and adults^18–23^, and an increasing application of skull base approaches in pediatric patients^18,19,24–31^. We herein perform a granular analysis of the IAC and surgically relevant parameters of the posterior petrous bone during normal pediatric maturation.

## Methods

### Study population

Retrospective review identified neurosurgical patients at Rady Children’s Hospital of San Diego and the University of California-San Diego during a 4-year period who underwent high resolution head computed-tomography (CT) imaging for focal cranial pathologies or trauma. Patients with traumatic fractures or other pathologies affecting the skull base, a history of hydrocephalus and/or a shunting procedure/endoscopic third ventriculostomy, or those with a CT scan not aligned to the skull base (as defined below) were excluded. Data was collected on a total of 50 randomly selected pediatric patients and 10 adult patients, out of a potential patient pool of more than 500 meeting the inclusion/exclusion criteria. Patients were evenly distributed by sex and the following ages groups: 0–3, 4–7, 8–11, 12–15, 16–18 years, and > 18 years, with an n of 10 patients per age category. These groupings were chosen to approximate developmental milestones including infancy/toddler (ages 0 to 3), early childhood (ages 4 to 7), middle childhood (ages 8 to 11), and adolescence (ages 12 to 18; split into pre- and postpuberty age groups).

The following variables were collected: age, sex, distance from the IAC porus acusticus to the fundus (IAC-L), IAC diameter at the porus (IAC-D), IAC diameter at the midpoint between the porus and fundus (IAC-DM), distance from the posterior aspect of the porus to the common crus (CC; P-CC), and the distance from the posterior petrosal surface (PPS) to the posterior semicircular canal (PSC; PPS-PSC).

### Internal auditory canal parameters

**Figure 1:**
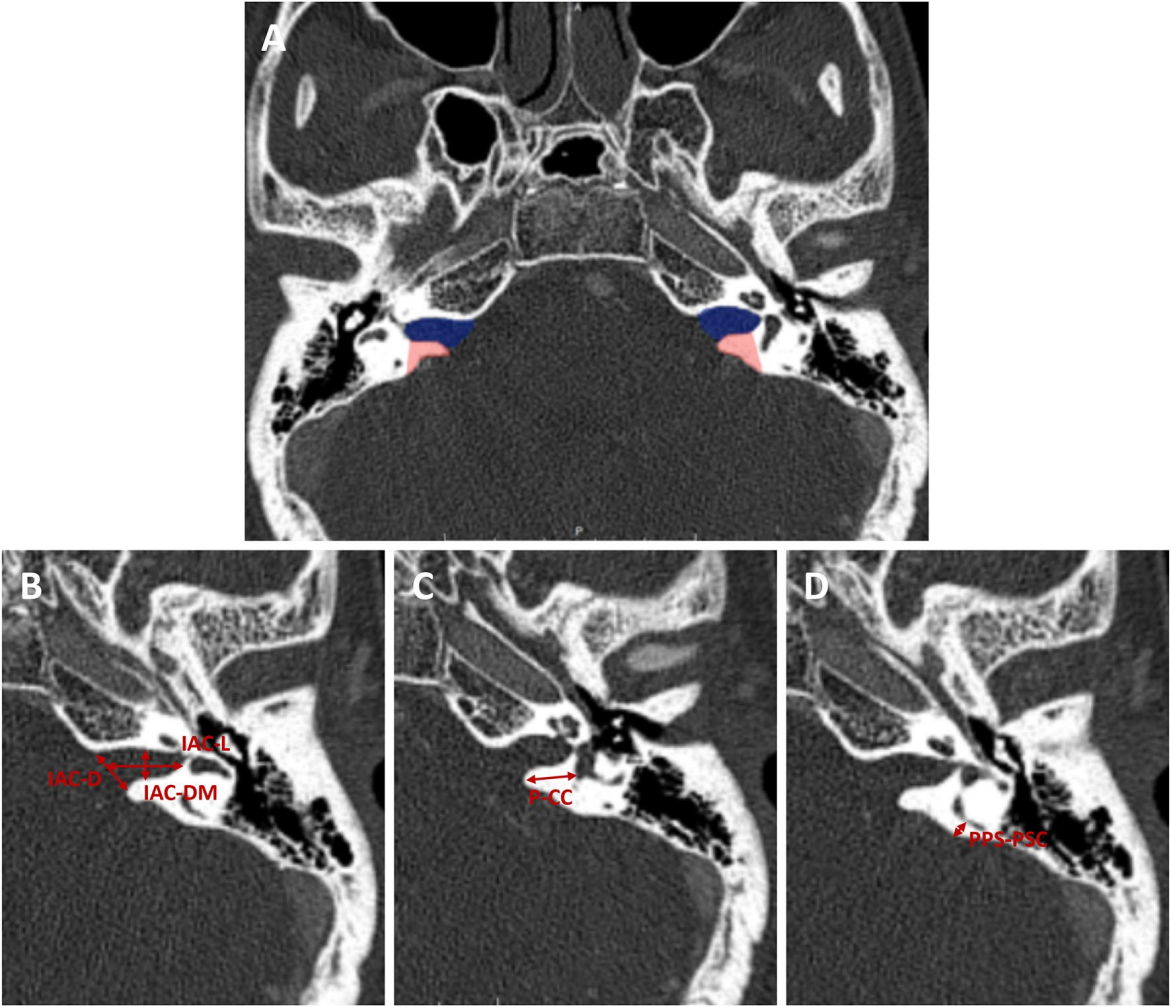
IAC and posterior petrous bone parameter measurements and surgical relevance. (**A**) Measurements of the IAC (highlighted in blue) and surgically relevant aspects of the posterior petrous bone were made from high-resolution CT scans. The area in red highlights bone removed during a retrosigmoid transmeatal approach, defined largely by the depth of the labyrinth from the posterior lip of the porous and the posterior petrosal surface (P-CC and PPS-PSC). (**B**) IAC length (IAC-L) was measured from the IAC porus to the fundus, along the center of the long axis of the IAC. IAC porus diameter (IAC-D) was measured from the anterior lip to the posterior lip of the IAC porus. IAC mid-point diameter (IAC-DM) was measured from the anterior to the posterior IAC at the midpoint between the porus and fundus (defined as the midpoint of IAC-L). (**C**) The distance from the porus to the common crus (P-CC) was measured from the posterior aspect of the IAC porus to the CC. (**D**) The distance from the posterior petrosal surface to the posterior semicircular canal (PPS-PSC) was measured from the posterior petrous face to the mid-aspect of the posterior semicircular canal.

IAC-L, IAC-D, and IAC-DM were measured on axial CT slices, using the slice with the largest visible cross-section of the IAC (Fig. 1). IAC-L was defined as the distance from the IAC porus (as marked by IAC-D) to the most lateral aspect of the fundus, along the center of the long axis of the IAC. IAC-D was defined as the distance from the most anterior to the most posterior aspect of the IAC porus. IAC-DM was defined as the anterior–posterior IAC diameter at the midpoint between the porus and fundus (defined as the midpoint of IAC-L).

IAC volume (IAC-V) was estimated based on the formula for a cone (volume = πr^2^ × height/3), with height defined by IAC-L and base radius by IAC-D/2. This volumetric approximation was based on our observations of IAC shape during data collection and prior studies suggesting the most common IAC morphology in children is cone-(or funnel) shaped^32^.

### Posterior petrous bone parameters

P-CC and PPS-PSC were measured on axial CT slices (Fig. 1). P-CC was defined as the distance from the posterior aspect of the IAC porus to the most mesial aspect of the CC. PPS-PSC was defined as the distance from the PPS to the mid-PSC.

### Measurements parameters

As in prior reports^21–23^, all measurements for analysis were made by a single author (R.C.R) after technique optimization on 10 CT scans from 10 excluded patients (data not included). Measurements were repeated on 10 random patients across a variety of ages by a second author (M.G.B.) to assess data reproducibility. Although gantry angles varied by scan based on patient head positioning, included head CTs were based on institutional conventions wherein axial slices were oriented parallel to the base of the skull and the supraorbital meatal line.

### Statistical analysis

Data on continuous variables is reported as mean ± standard deviation (SD) or standard error of the mean (SEM). IAC and posterior petrosal bone parameters were analyzed by laterality, sex, and age using paired t-tests, unpaired t-tests, and linear regression. Age was considered a categorical variable, broken into six groups (0–3, 4–7, 8–11, 12–15, 16–18 years, and > 18 years). After testing for significant differences by laterality, left and right data for individual patients were averaged to reduce statistical comparisons. Corrections for multiple comparisons were made to reduce the likelihood of Type 1 error using the Student–Newman–Keuls (SNK) method. Scatterplots of standardized fit parameters by age group (obtained by subtracting the mean and dividing by the SD) were compared via regression line coefficient analysis by using Stata’s “suest” postestimation command. To assess for differences in measurements by author (R.C.R. versus M.G.B.), paired two sample t-tests and Pearson correlation coefficients were calculated between datasets (after left and right data were averaged). All statistical analyses were performed using Stata MP Version 14.1 (StataCorp LP). Statistical significance was defined as a p-value < 0.05.

### Consent

All methods were performed in accordance with relevant guidelines and regulations of the Rady Children’s Hospital-San Diego/University of California-San Diego Institutional Review Boards (IRBs) and Health Insurance Portability and Accountability Act. The Rady Children’s Hospital-San Diego/University of California-San Diego Institutional Review Board (IRBs) approved this study, including waiver of individual consent for collection and analysis of the retrospective de-identified data in this work. Individual patient consent for procedures was obtained for all patients.

## Results

By group, mean ± SD ages were: 2.0 ± 1.0 years (ages 0–3); 5.2 ± 1.1 years (ages 4–7); 9.4 ± 1.3 years (ages 8–11); 12.9 ± 1.0 years (ages 12–15); 17.1 ± 0.6 years (ages 16–18); and 49.1 ± 15.0 years (adult). IAC and posterior petrosal bone measurements largely displayed no significant differences by laterality (Table 1) or sex (Table 2), with the exception of IAC-DM and P-CC being greater on the left.

**Table 1:** Internal auditory canal and posterior petrous bone parameters by laterality.

Parameter	N	Left (mean)	Right (mean)	Mean difference	p
IAC-L	60	10.24	10.34	− 0.09	0.528
IAC-D	60	7.83	7.92	− 0.09	0.582
IAC-DM	60	4.74	4.53	0.21	0.014
IAC-V	60	168.31	175.98	− 7.67	0.345
P-CC	60	9.41	9.10	0.30	0.031
PPS-PSC	60	2.87	3.01	− 0.14	0.184

Significance determined by paired t-tests. All parameters are in mm, except IAC-V (mm^3^).

**Table 2:** IAC and posterior petrous bone parameters by sex.

Parameter	Female	N	Male	N	Mean difference	p
IAC-L	10.25	30	10.33	30	− 0.08	0.838
IAC-D	7.79	30	7.96	30	− 0.17	0.562
IAC-DM	4.57	30	4.69	30	− 0.12	0.546
IAC-V	169.63	30	174.67	30	− 5.04	0.725
P-CC	9.37	30	9.14	30	0.22	0.622
PPS-PSC	3.16	30	2.72	30	0.44	0.200

Significance determined by independent-samples t-tests. All parameters are in mm, except IAC-V (mm^3^).

**Figure 2:**
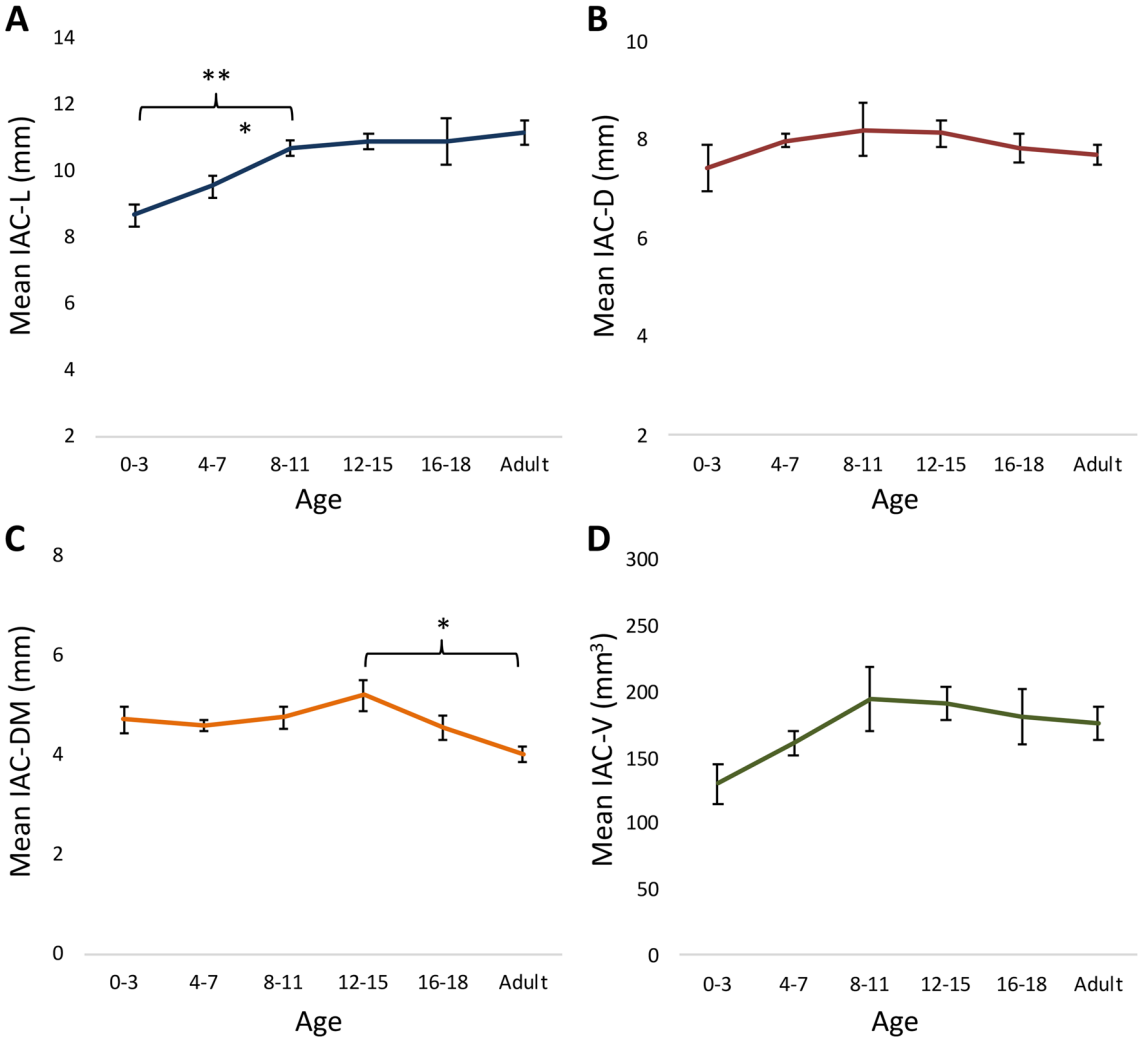
Maturation of the IAC. (**A**) IAC-L increased 27.5% from 8.7 ± 1.1 at age 0–3 to 11.1 ± 1.1 mm at adulthood (p = 0.001). The majority of this growth occurred between the ages of 0–3 and 8–11, before leveling off. (**B**) IAC-D, (**C**) IAC-DM, and (**D**) IAC-V did not display significant changes from ages 0–3 to adulthood. IAC-DM decreased 23.1% from ages 12 to 15 (5.2 ± 1.0 mm) to adulthood (4.0 ± 0.5 mm). The IAC-L and IAC-DM data suggest an elongation and narrowing of the IAC over time. *Indicates p < 0.05 and **indicates p < 0.01 on linear regression.

**Figure 3:**
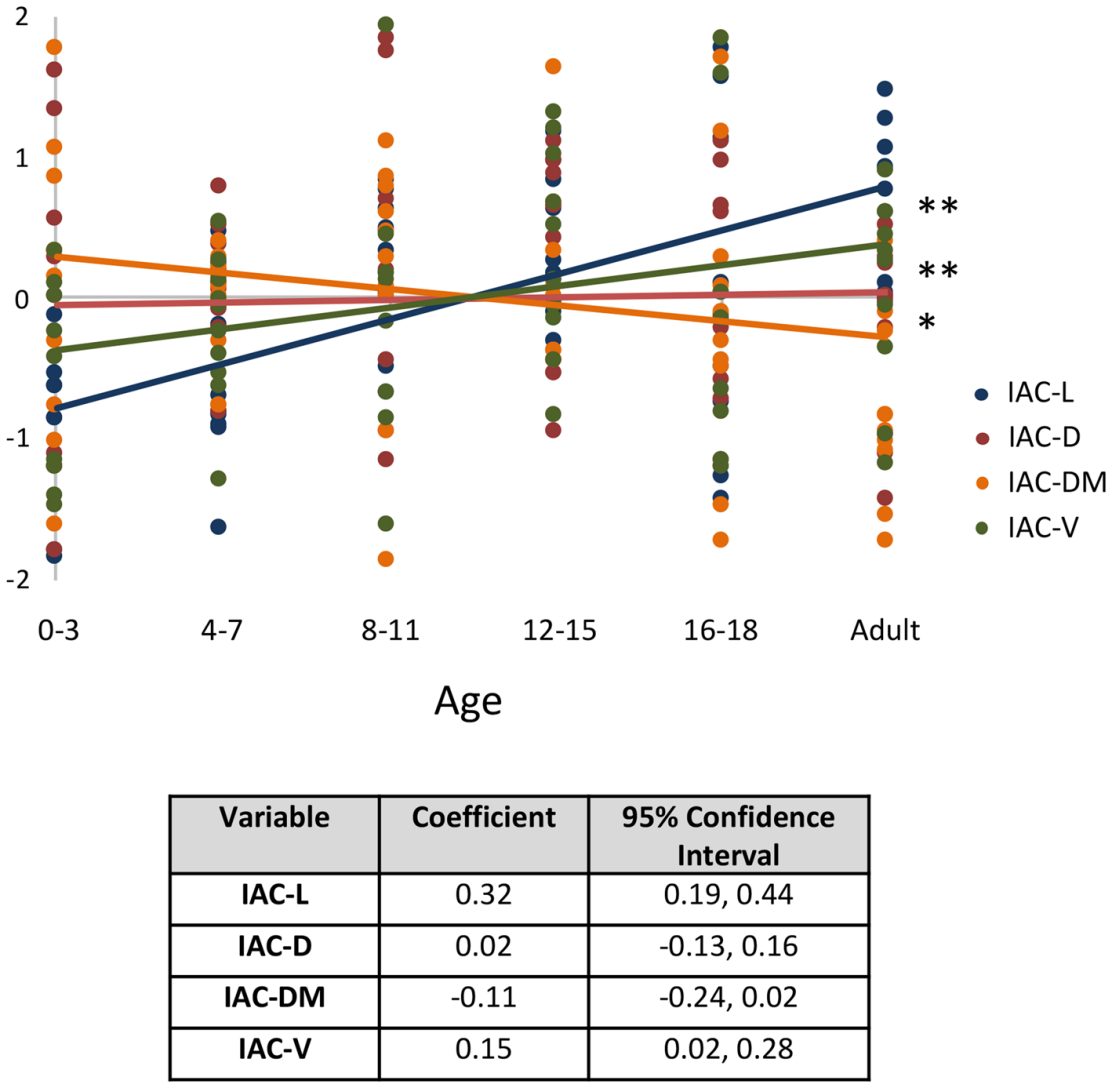
Standardized IAC growth curves. Normalized scatterplot and linear regression analysis demonstrated significant differences between the IAC-L, IAC-D, IAC-DM, and IAC-V growth rates. *Indicates p < 0.05 and **indicates p < 0.01.

Regarding IAC parameters, IAC-L displayed the most notable changes with maturation, increasing 27.5% from 8.7 ± 1.1 at age 0–3 to 11.1 ± 1.1 mm at adulthood (p = 0.001) (Table 3). The majority of this growth occurred between the ages of 0–3 and 8–11 (p = 0.002), reaching interval significance from ages 4–7 to 8–11 (p = 0.042) (Fig. 2A). Although IAC-D did not display significant changes either from ages 0–3 to adulthood (p = 0.52), or across consecutive age groups, it did reach a peak of 8.2 ± 1.7 mm at ages 8–11 (Fig. 2B). IAC-DM also did not display significant changes from ages 0–3 to adulthood (p = 0.167), or across consecutive age groups, but did display a 23.1% decrease in size from a peak at ages 12–15 (5.2 ± 1.0 mm) to adulthood (4.0 ± 0.5 mm) (p = 0.007) (Fig. 2C). While IAC-V did not demonstrate significant changes either from ages 0 to 3 to adulthood (p = 0.132), or across consecutive age groups, a trend toward growth from 0–3 to 8–11 (49.8%, from 129.9 ± 46.9 to 194.6 ± 75.6 mm^3^; p = 0.081) was seen before leveling off (Fig. 2D). Standardized fit curves demonstrated significant differences between the IAC-L, IAC-D, IAC-DM, and IAC-V growth rates, with IAC-DM notable for a negative slope during the course of pediatric development (Fig. 3).

**Table 3:** IAC parameters by age (years).

Parameter, mean (SD)	0–3	p	4–7	p	8–11	p	12–15	p	16–18	p	Adult	p*
N	10	–	10	–	10	–	10	–	10	–	10	–
IAC-L	8.7 (1.1)	0.131	9.5 (1.0)	0.042	10.7 (0.8)	0.756	10.9 (0.7)	0.986	10.9 (2.2)	0.638	11.1 (1.1)	0.001
IAC-D	7.4 (1.5)	0.697	8.0 (0.5)	0.908	8.2 (1.7)	0.905	8.1 (0.8)	0.799	7.8 (0.9)	0.819	7.7 (0.7)	0.605
IAC-DM	4.7 (0.8)	0.711	4.6 (0.3)	0.866	4.7 (0.7)	0.163	5.2 (1.0)	0.250	4.5 (0.8)	0.119	4.0 (0.5)	0.167
IAC-V	129.9 (46.9)	0.198	160.5 (28.7)	0.598	194.6 (75.6)	0.880	191.0 (39.8)	0.671	181.0 (69.1)	0.827	175.9 (38.2)	0.132
P-CC	7.7 (1.5)	0.098	8.9 (1.6)	0.562	9.3 (1.2)	0.807	9.8 (1.5)	0.994	9.8 (2.2)	0.903	10.1 (1.5)	0.021
PPS-PSC	1.5 (0.7)	0.068	2.6 (1.2)	0.703	2.4 (0.6)	0.135	3.3 (0.8)	0.234	3.9 (1.5)	0.932	3.9 (1.2)	< 0.001

Pairwise p-values between adjacent groups corrected using SNK method.

*Indicates p-value for 0–3 to adult. All parameters are in mm, except IAC-V (mm^3^).

**Figure 4:**
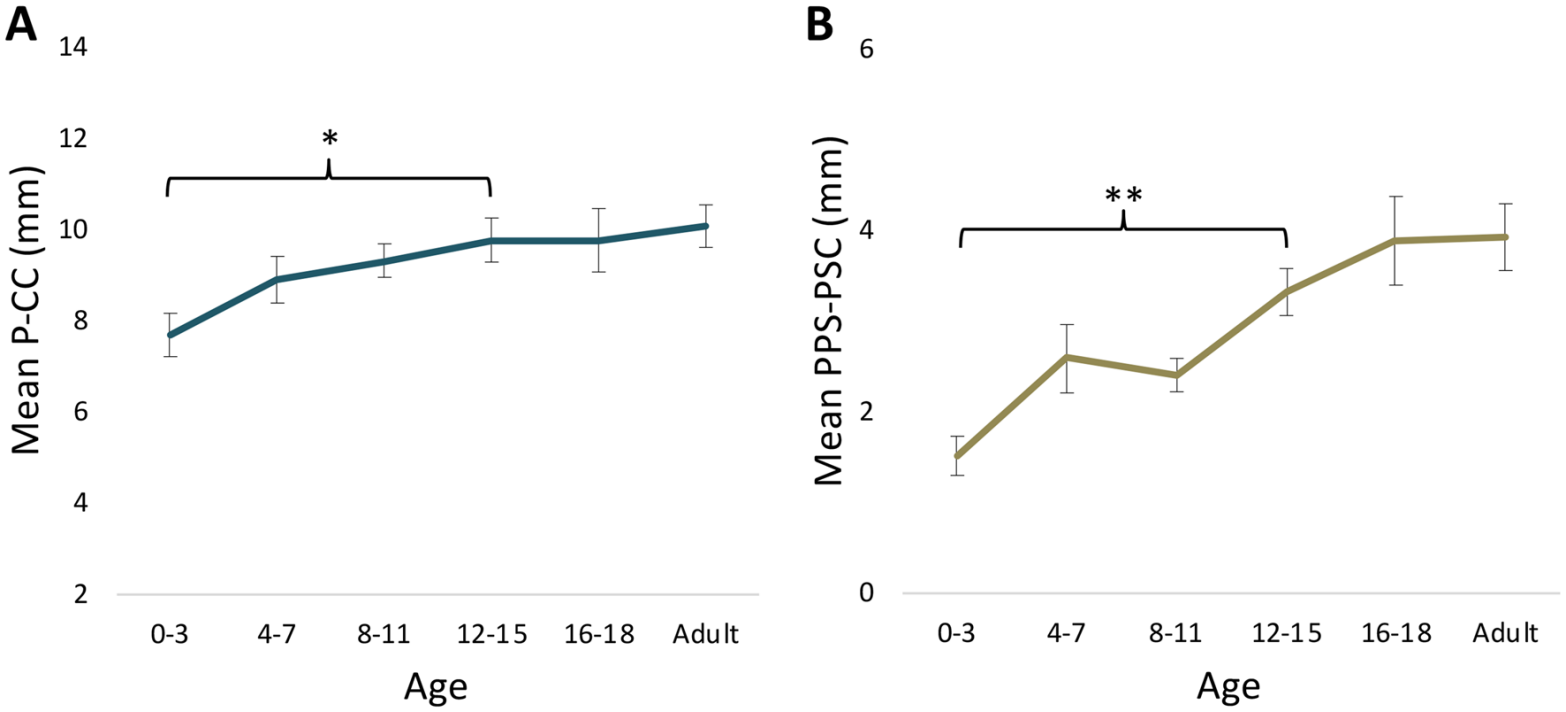
Maturation of the posterior petrous bone. (**A**) P-CC increased 31.1% from 7.7 ± 1.5 at age 0–3 to 10.1 ± 1.5 mm at adulthood (p = 0.019). (**B**) PPS-PSC increased 160% from 1.5 ± 0.7 at age 0–3 to 3.9 ± 1.2 mm at adulthood (p < 0.001). The majority of growth for both P-CC and PPS-PSC occurred from ages 0–3 to 12–15, before leveling off.

**Figure 5:**
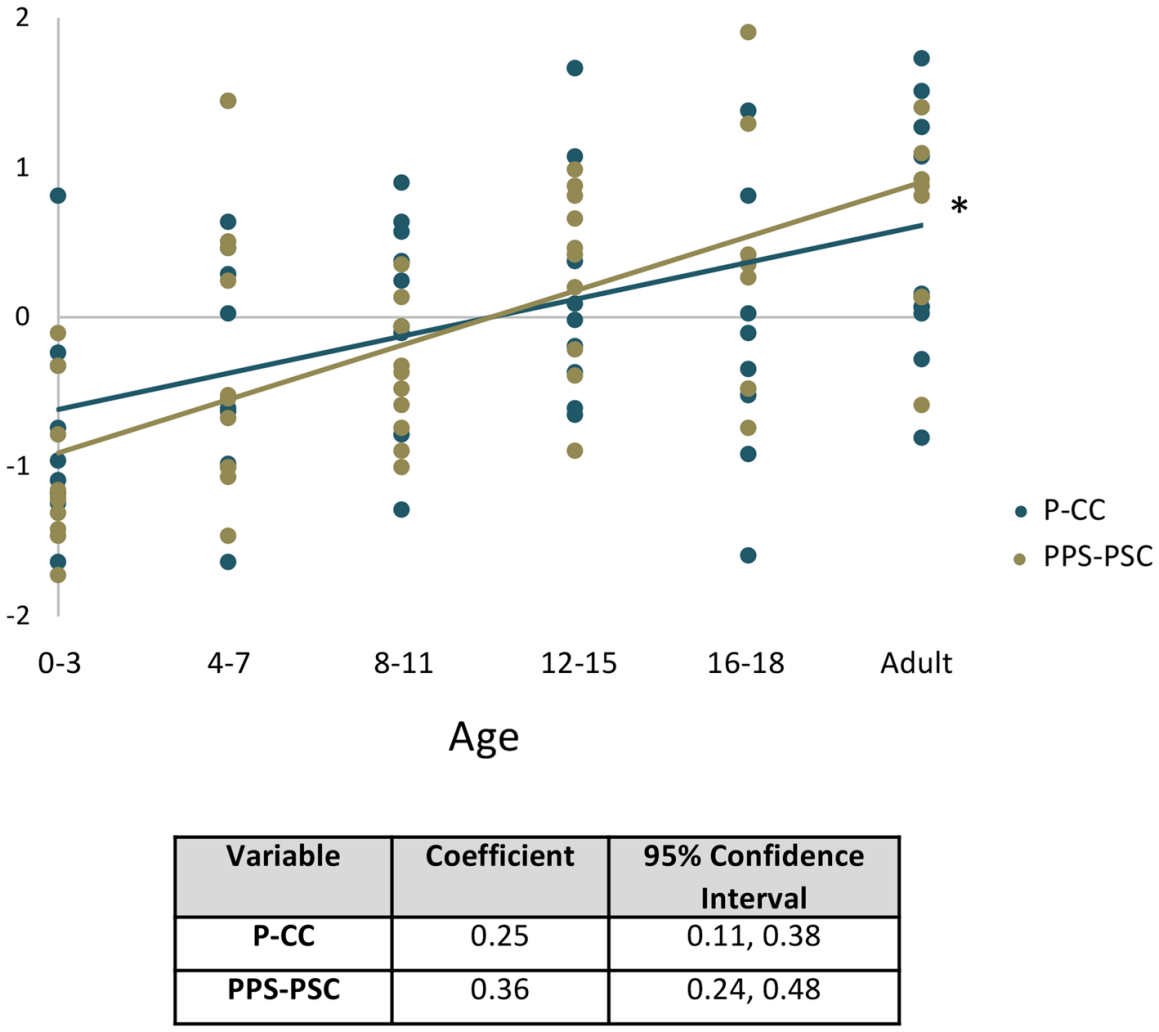
Standardized posterior petrous growth curves. Normalized scatterplot and linear regression analysis demonstrate a higher growth rate of PPS-PSC as compared to P-CC. *Indicates p < 0.05.

The posterior petrous bone parameters P-CC and PPS-PSC displayed growth patterns most similar to IAC-L with maturation. P-CC increased 31.1% from 7.7 ± 1.5 at age 0–3 to 10.1 ± 1.5 mm at adulthood (p = 0.019), with the majority of this growth occurring from ages 0–3 to 12–15 (p = 0.042) (Table 3, Fig. 4A). PPS-PSC increased 160% from 1.5 ± 0.7 at age 0–3 to 3.9 ± 1.2 mm at adulthood (p < 0.001), again with the majority of this growth occurring from ages 0–3 to 12–15 (p = 0.002) (Fig. 4B). Standardized fit curves confirmed PPS-PSC had a significantly higher growth rate with maturation as compared to P-CC (p = 0.044) (Fig. 5).

Supporting the fidelity of this data, no significant differences in any parameter were seen between the primary and test datasets as measured by different authors, with Pearson correlations for all parameters > 0.8.

## Discussion

The known variability of the IAC and the petrous temporal bone has important surgical implications. For example, the orientation and position of the IAC within the petrous bone can significantly affect visualization of the facial nerve from a translabrynthine approach^1^. The length of the IAC and distance from the IAC porus to the common crus, as well as the depth of the posterior semicircular canal from the surface of the posterior petrous bone can similarly affect the maximum safe exposure of the IAC via a retrosigmoid transmeatal approach (Fig. 1A)^2^. The length and diameter of the IAC is also relevant to more recently described techniques, such as the minimally invasive expanded transcanal transpromontorial approach to the IAC through the external auditory canal^33,34^. Conversely, knowledge of structures that are relatively constant, such as the relationships between the cochlea, superior semicircular canal, and the facial nerve at the fundus of the IAC is critical to optimize exposure of the IAC while preserving hearing with a middle fossa approach^35^. An awareness of how the anatomy of this region changes with maturation is a pre-requisite for the performance of these skull base approaches in children.

This work builds on our prior studies detailing the surgical implications of maturation of the occipital condyle, jugular tubercle (JT), anterior clinoid process (ACP), and anterior petrous apex (APA)^21–23^, In line with our previous findings, maturation of the IAC and posterior petrous bone is nonlinear. Unlike the biphasic growth pattern of the JT^21^, or the early growth then plateau of the APA^23^, the development of the IAC most closely resembles the dynamic elongation and remodeling seen with the ACP^22^. The increase in IAC-L up until ages 8–11, combined with the stability of IAC-D and decrease in IAC-DM from ages 12 to 15 to adult, effectively remodels the IAC from a short, wide cone to a longer, narrower canal over time, while keeping its net volume relatively stable. The maturation of the posterior petrous bone is more straightforward, with P-CC and PPS-PSC demonstrating concentrated growth from birth until early adolescence, reaching near-adult dimensions by ages 12–15, with little growth thereafter. Highlighting the variations in skull base development by region, the growth of the measured posterior petrosal dimensions is slightly prolonged as compared the APA region, which aside from the depth of the IAC from the middle fossa floor is largely completed by ages 8–11^23^. It is nonetheless completed earlier than the final maturation of the JT, which occurs by ages 16–18^21^, or the ACP which matures up until adulthood^22^.

Supporting our methodology, the parameters measured in the adult control group in this study are in line with previous adult cadaveric and imaging-based works. Specifically, our adult IAC-L of 11.1 ± 1.1 mm fits within the 9.8 to 11.6 mm reported means (range 6–16.5 mm) of prior studies^2,9–15,32^, and our adult IAC-D (7.7 ± 0.7 mm) and IAC-DM (4.0 ± 0.5 mm) measurements closely match prior reports (mean 6.2 to 7.5, range 2.6–12.4 mm for IAC-D and mean 4.2 to 6.5, range 2.3–8.1 for IAC-DM)^2,10,13,14,32,36^. Our adult posterior petrous measurements P-CC (10.1 ± 1.5 mm) and PPS-PSC (3.9 ± 1.2 mm) are also supported by prior dissection and imaging-based temporal bone analyses (mean 7.5 to 10.4 mm, range 5.3–13.5 mm for P-CC and mean 3.3 to 3.6 mm, range 0.6–6.5 mm for PPS-PSC, despite variations in measurement technique for the latter parameter)^2,3,13^. The wide ranges reported within these prior works nonetheless underscores the variability possible with this anatomy.

Although studies of the IAC and posterior petrous bone in pediatric populations are more limited, our data largely fit with prior observations and increase the granularity of our understanding of the development of these structures. In a study by Bonaldi et al., IAC molds from 98 dried temporal bones on subjects ranging from 7-month-old fetuses to adults were grouped (I: 7–9-month fetuses, II: 2–11 month old infants, III: 1–6 year old children, and IV: adults) and measured^16^. Despite a focus on the prenatal, infancy and early childhood periods (≤ 6 years of age) in this study, a similar elongation of IAC-L (measured along the anterior, posterior, superior, and inferior walls) and overall stability of IAC-DM was observed (with the narrowing of IAC-DM from ages 12 to 15 to adulthood in our study outside of the analyzed populations). The reported increase in IAC-D (notably in the axial but not coronal plane) in this work (from 4.02 in group I to 7.03 mm in group IV) was nonetheless concentrated within groups I–III, indicative of an early widening of the porous in the late prenatal and early post-natal period. This growth was already largely present in the 0–3 age group in our study (mean 7.4 ± 1.5 mm versus a mean of 6.09 ± 0.92 mm in group III in Bonaldi et al.), before leveling off throughout the rest of development. IAC-D growth that stopped by approximately 1 year of age and an overall stability of IAC-DM throughout development was also reported by Sakashita and Sando in a study of 20 temporal bones from subjects aged 1 month to 72 years^17^, with the narrowing of the IAC-DM during later development in our data potentially not captured by the small n of this study. Although it is unclear why the observed changes in IAC-DM lag behind IAC-L maturation, it is conceptually consistent with the conclusion reached by Sakashita and Sando that the IAC develops mainly along its long axis away from the early matured otic capsule (elongating and potentially narrowing the medial canal over time)^17^. Further supporting our findings, these authors report a similar, significant increase in IAC-L from birth to age 10, after which no further growth was noted through adulthood. Sakashita and Sando also report IAC-V using computer aided reconstructions, with their volumetric growth curve and maturation by age 10 closely matching our data. Although IAC-V growth during development reached significance in the Sakashita and Sando study (compared to the near-significant trends observed in our data), this difference is likely driven by concentrated volumetric growth within the first year of life that was not granularly captured herein.

Our results differ somewhat from the findings of Marques et al., who studied IAC morphology using CT scans of 110 subjects across an age range of 1 to 92 years^32^. When comparing the IACs of children (up to 14 years) and adults (19 to 92 years) in this work, IAC-D was largely stable and IAC-DM decreased with maturation, mirroring our data. However, rather than elongation Marques et al. report a decrease in IAC-L during development, with a mean of 11.17 mm in children to 9.84 mm in adults. The mean and distribution of ages within these cohorts is nonetheless not reported in this work, and given the maturation to near adult parameters of IAC-L in our data by ages 8–11, if a relatively older cohort of children was analyzed the IAC-L would be expected to be closer to adult size (supported by the 11.17 mm mean IAC-L in children in Marques et al. resembling the 11.1 mm mean IAC-L in adults in our study, and the 9.84 mm mean IAC-L in adults in Marques et al. representing the low end of previous analyses)^2,9–15,32^. To the best of our knowledge, prior granular data on the effects of maturation on posterior petrous bone parameters were not available for comparison.

These data, combined with a close pre-operative imaging review, may aid the selection and performance of surgical approaches to lesions near to or involving the IAC in pediatric patients. For example, in the older pediatric population where the anatomic dimensions of the IAC and surrounding structures are largely adult size by ages 12–15^23^, few modifications may be needed in the performance of skull base approaches to this region as compared to adults. Conversely, in younger patients, awareness of a shorter IAC-L, P-CC, and PPS-PSC is important to avoid an inner ear breach during exposure of the IAC via a middle fossa or retrosigmoid transmeatal approach. The relative proportion of the IAC available for safe exposure is also smaller in this population, as IAC growth occurs preferentially medially to the foramen singulare, a surgical landmark of the labyrinth during a retrosigmoid transmeatal exposure of the IAC^11,17^. The predominately open conical shape and largely preserved volume of the IAC in younger patients may nonetheless aid visualization of the proximal IAC despite these constraints. Accordingly, in our experience the safe performance of these approaches is possible, even in patients less than 2 years old^27,31^.

A limitation of this work is the use of imaging-based rather than cadaveric measurements. Prior works have nonetheless demonstrated the comparability of these approaches for studying this region^2^. Obtaining cadaveric specimens of adequate numbers within each of the selected age groups was also impractical. As described in previous studies using a similar methodology by our group^21–23^, multiple steps including the use of strict patient inclusion/exclusion criteria and statistical analyses to minimize selection biases were taken to optimize the fidelity of this data. To limit interrater variability, all measurements used for analysis were performed by a single author after a training period for familiarization with variations in bony anatomy across patients. Supporting the accuracy of our data, repeat measurements on randomly selected patients across age groups by a second author demonstrated no significant differences and a high level of correlation between authors.

## Conclusion

Maturation of the IAC and surrounding region is largely complete by ages 12–15. Awareness of the growth patterns of this area may facilitate the safe performance of skull base approaches to the IAC in pediatric patients, including the hearing-preserving middle fossa and retrosigmoid transmeatal approaches.
